# Biomass-Derived Laser-Induced Graphene/Chitosan Composite Films for Sustainable Triboelectric Nanogenerators

**DOI:** 10.3390/nano16090550

**Published:** 2026-04-30

**Authors:** Chong Chen, Zhenyuan Chui, Yaokun Pang

**Affiliations:** Shandong Key Laboratory of Renewable Membrane Materials, College of Materials Science and Engineering, Qingdao University, Qingdao 266071, China; 18391058513@163.com (C.C.); chuizy1109@126.com (Z.C.)

**Keywords:** triboelectric nanogenerator, chitosan, laser-induced graphene, biomass

## Abstract

As a green energy technology, triboelectric nanogenerators (TENGs) convert mechanical energy into electricity and have gained significant attention in response to growing global environmental concerns. However, the widespread use of petroleum-based polymers as triboelectric materials in high-performance TENGs raises concerns over plastic pollution. In this work, we report a high-performance biodegradable TENG utilizing chitosan/laser-induced graphene (LIG) composite films as triboelectric layers. Modified chitosan substrates were first converted into LIGs via a convenient one-step CO_2_ laser engraving, subsequently incorporated into chitosan matrices to form homogeneous composite films. A TENG device was designed by pairing the LIG/chitosan composite film with the fluorinated ethylene propylene (FEP) film, and copper electrodes. The introduction of LIG effectively strengthens charge storage and dielectric properties of the chitosan matrix, thereby significantly boosting the triboelectric output performance. Experimental results demonstrate that the as-assembled TENG with an LIG concentration of 1 wt.% achieves a peak open-circuit voltage of 196 V and short-circuit current of 2.1 μA, with a maximum power density of 295 mW/m^2^. It can drive LED lights and small low-power electronic devices. Furthermore, the designed TENG device exhibits good biodegradability, flexibility, and stability, serving as a self-powered sensor for monitoring human joint movements. This work provides a simple and scalable strategy for integrating laser-induced graphene with biomass-based polymers, offering new insights into the design of high-performance, biobased triboelectric materials.

## 1. Introduction

In recent years, the rapid advancement of flexible electronics has led to their increasingly widespread application across diverse sectors, including health monitoring, wearable sensing systems, human–machine interaction, and energy harvesting [1,2,3,4]. However, these devices currently rely primarily on traditional chemical batteries for power, posing potential threats to the natural environment and ecosystems. In the face of increasingly severe environmental pollution challenges, the development of novel clean alternative energy sources has become an urgent priority [5]. Against this background, the triboelectric nanogenerator (TENG) has emerged as a promising clean energy harvesting technology, providing an ideal solution to replace chemical batteries and power flexible electronic devices. Based on the coupling of contact electrification and electrostatic induction, TENGs can directly convert various forms of mechanical energy from the environment into electricity [6,7]. Furthermore, TENGs possess significant advantages, such as compact dimensions, lightweight nature, high structural conformability, environmental friendliness, and stable electrical output [8].

Currently, the fabrication of most TENGs still relies on non-degradable synthetic polymers [9,10,11] such as polyethylene terephthalate (PET), polydimethylsiloxane (PDMS), and polyimide (PI). These materials not only consume the nonrenewable petroleum-based resources but also are difficult to degrade naturally after disposal, and the resulting electronic waste management issues significantly hinder the further application and advancement of TENG technology [12]. To overcome these limitations, researchers have turned their attention to abundant, renewable, and biocompatible natural biomass materials. Among numerous biopolymers, chitosan (CS) has demonstrated immense potential in the development of high-performance, eco-friendly triboelectric layer materials due to its unique molecular structure and excellent physicochemical properties. As the second most abundant natural polymer after cellulose, chitosan is recognized as one of the most valuable natural polymers owing to its inherent antibacterial, antifungal, and antiviral properties, as well as its complete biocompatibility, biodegradability, and non-toxicity. Additionally, it exhibits outstanding film-forming, fiber-forming, and hydrogel-forming capabilities. Thus, CS is widely recognized as one of the most promising biobased materials for TENG triboelectric layers [13]. At present, the application of CS-based TENGs in the field of wearable sensors has become a focal point of intensive research [14].

Despite the advantages of biodegradable polymers as premium triboelectric layer materials, pure biomass-based TENGs still face significant performance bottlenecks, largely constrained by the intrinsic limitations of natural materials. Specifically, these limitations include low surface charge density, inherent brittleness, and high sensitivity to environmental humidity, which collectively hinder their practical application in high-performance self-powered systems [15,16,17,18]. Therefore, enhancing performance output while maintaining complete biodegradability has become a core challenge in the field of flexible electronics. To enhance triboelectric performance, incorporating heterogeneous fillers is essential. For instance, Supakit et al. incorporated AgNPs into cellulose fibers (CF) to form CF@Ag hybrid fillers, which were then embedded into a natural rubber matrix, significantly boosting the power output of the resulting TENGs [19]. Similarly, Jin et al. utilized ZnO to modify chitosan/chondroitin sulfate composite films for the fabrication of flexible TENGs [20]. Other approaches include the work of Kaur et al., who synthesized TENGs by compositing SnO nanoparticles onto waste biomaterials such as eggshell membranes (ESM) [21], and Wang et al., who employed NH_2_-MXene/TiO_2_ to modify sodium alginate for corrosion-resistant flexible TENGs [22]. However, these strategies inevitably introduce non-degradable metal oxides, which undermines the environmental benefits of the resulting electronics. Graphene is an effective filler [23], but traditional synthesis methods like CVD are hindered by high costs [24]. Laser-induced graphene (LIG) offers a cost-effective alternative for graphene synthesis. Notably, chitosan can serve as both a high-quality triboelectric matrix and an excellent carbonaceous precursor for biomass-derived LIG [25], owing to its carbon-rich molecular framework and specific functional groups (such as hydroxyl and amino groups) that facilitate efficient photothermal conversion. This dual-functionality provides a unique opportunity for achieving material homogeneity within the composite system.

Herein, we report a high-performance, fully biodegradable TENG based on laser-induced graphene/chitosan/glycerol (LCG) composite films prepared via a homologous conversion strategy. By incorporating aminotrimethylene phosphonic acid (ATMP), the chitosan-based precursor achieves stable one-step laser graphitization and enhanced flame retardancy while maintaining excellent mechanical integrity. This process enables the precise fabrication of LIG as a functional filler within a localized CS matrix. Systematic optimization reveals that the LCG composite film with 1% LIG content delivers peak output performance, including an open-circuit voltage (V_OC_) of 196 V, a short-circuit current (I_SC_) of 2.1 μA, and a power density of 295 mW/m^2^. Beyond its robust electrical properties, the LCG-TENG exhibits complete environmental degradability in both H_2_O_2_ and pepsin solutions. During physical activity, the device effectively harnesses biomechanical energy from various human movements to trigger contact-separation between LCG and FEP layers, generating discrete electrical signals for real-time motion perception. This work effectively addresses the trade-off between high-performance output and full biodegradability, providing a versatile and sustainable platform for next-generation wearable electronics.

## 2. Materials and Methods

### 2.1. Materials

All chemical reagents employed in this study were used as received without further purification. Chitosan (deacetylation ≥ 90%), glycerol (AR, 99%), and acetic acid (AR, 99.5%) were purchased from Macklin (Shanghai, China). Amino trimethylene phosphonic acid (ATMP, 99%) was obtained from Tianjin Xeowns Biochemical Technology Co., Ltd. (Tianjin, China). Additionally, pepsin and hydrogen peroxide (H_2_O_2_, 3 wt.% in H_2_O, containing ~200 ppm acetanilide as a stabilizer) were supplied by Shanghai Aladdin Biochemical Technology Co., Ltd. (Shanghai, China).

### 2.2. Preparation of CS-ATMP Films

The CS-ATMP composite films were fabricated via a solution casting method. Initially, 1 g of CS powder was added to 30 mL of acetic acid (2% *v*/*v*) to obtain a CS solution. The mixture was stirred continuously in a water bath at 30 °C for 5 h to yield a viscous, yellowish, and transparent CS solution. Subsequently, amino trimethylene phosphonic acid (5 wt.%) was incorporated into the CS solution, followed by stirring at 30 °C for 3 h to achieve a homogeneous mixture. The resulting solution was then degassed for 5 h until no air bubbles were visible. Finally, the uniform CS-ATMP solution was transferred to a Petri dish and dried at 60 °C for 4 h to yield the final CS-ATMP composite films.

### 2.3. Preparation of Powdered LIG

The LIG structures were fabricated via laser irradiation using a commercial CO_2_ laser engraving system (KB-4060, Liaocheng Kebai Laser Equipment Co., Ltd., Liaocheng, China) equipped with a 10.6 μm wavelength laser and a rated power of 60 W. The LIG was synthesized under specific conditions: a laser power of 4.7 W and a scanning speed of 12 mm/s. Square and rectangular geometries were initially designed in Corel DRAW X4 SP2. Subsequently, the patterns were created on the surface of the CS-ATMP films through raster scanning of the laser beam. Upon completion of the engraving process, the top-layer LIG was exfoliated from the substrate and ground to obtain the final LIG powder.

### 2.4. Preparation of LCG Composite Films

The LCG composite films were fabricated via a solution casting method. Initially, LIG powder was dispersed in 30 mL of acetic acid solution (2% *v*/*v*) at specific mass fractions (0 wt.%, 0.5 wt.%, 1 wt.%, 2 wt.%, 3 wt.%, and 4 wt.%). The dispersion was subjected to ultrasonic treatment using an ultrasonic cell crusher (LC-CP900, Weifang Laneade Instrument Co., Ltd., Weifang, China) equipped with a No. 3 horn at an operating power of 100 W. Subsequently, 1 g of chitosan (CS) powder and 0.3 g of glycerol were added to the mixture, which was stirred continuously in a water bath at 30 °C for 5 h to obtain a viscous CS-glycerol-LIG solution, varying in color from light gray to black depending on the LIG loading. The resulting composite solution was degassed by standing for 24 h at room temperature. Finally, the solution was cast and dried in an oven at 40 °C for 24 h to yield the LCG composite films as detailed in Table 1.

### 2.5. Fabrication and Performance Testing of LCG-Based TENGs

To implement a vertical contact-separation mode TENG, an LCG-based TENG was designed, utilizing the LCG film as the tribopositive layer and an FEP film as the tribonegative layer. Copper foils were employed as electrodes, and the total effective area of the device was 5 × 5 cm^2^. The TENG was driven by a linear motor to maintain periodic contact and separation. The resulting electrical outputs, including short-circuit current, transferred charge, and open-circuit voltage, were characterized and recorded using a programmable electrometer (Keithley 6514 System Electrometer, Keithley Instruments, LLC, Beaverton, OR, USA). The results showed that when the content of LIG was 1%, the LCG composite film had the highest output performance with an open-circuit voltage (Voc) of 196 V, a short-circuit current (Isc) of 2.1 μA, a transferred charge of 82 nC, and a power density of 295 mW/m^2^.

### 2.6. Degradation Tests

The degradation profiles of the LCG composite films (1 wt.% LIG loading, dimensions of 1 × 1 cm^2^) were investigated by separately immersing the samples in two distinct media: an H_2_O_2_ solution at ambient temperature and a 1% pepsin solution maintained at 37 °C. The physical changes and degradation progress of the films were systematically observed and recorded over time.

### 2.7. Characterization and Measurement

The surface morphology and elemental distribution of the CS-LIG composite films (with LIG on the surface) were characterized using field-emission scanning electron microscopy (FE-SEM, Quanta 250 FEG, FEI Company, Hillsboro, OR, USA). For SEM observation, samples were cut into 1 × 1 mm^2^ rectangles and secured onto the sample stage using conductive carbon tape. To enhance surface conductivity and prevent charging effects, the specimens were sputter-coated with a thin layer of gold. The chemical states and elemental compositions were analyzed via X-ray photoelectron spectroscopy (XPS, PHI 5000 Versa Probe III, ULVAC-PHI, Inc., Chigasaki, Kanagawa, Japan). For XPS characterization, a small amount of LIG powder was compressed into a 3 mm^2^ pellet using a tablet press (YLJ-100M, Hefei Kejing Materials Technology Co., Ltd., Hefei, Anhui, China), while CSG films were prepared as 3 × 3 mm^2^ specimens; both were fixed onto the sample holder using double-sided adhesive tape. Raman spectroscopy (Thermo Fisher DXR2, Waltham, MA, USA) was conducted to evaluate the graphitization degree. LIG powder and 1 × 1 mm^2^ CSG film samples were placed on the stage, with the laser probe carefully aligned with the sample surface for measurement over a spectral range of 500–2500 cm^−1^. Additionally, the optical properties were analyzed using a UV-Vis-NIR spectrophotometer (UV-3600Plus, Shimadzu, Kyoto, Japan) within a wavelength range of 200–2500 nm. For these measurements, LCG composite films were prepared as 1.5 × 3 cm^2^ specimens and mounted directly onto the film holder. Furthermore, the surface morphology of the composite films was observed using optical microscopy (Leica DM2700P, Wetzlar, Germany) to further analyze their macrostructural features.

### 2.8. Mechanical and Electrical Performance Testing

Mechanical properties of the LCG composite films with varying LIG loadings were evaluated using a universal tensile testing machine (In 5300, Instron Corp, Norwood, MA, USA). The samples were prepared as 4 cm × 3 mm rectangular strips with a notched (chamfered) design in the middle to concentrate stress and ensure consistent fracture. The specimens were then securely fixed in the grips for tensile testing.

### 2.9. Electrical Performance Measurements

The output performance of the LCG-based TENGs was evaluated in a vertical contact-separation mode. The device, featuring a total effective contact area of 5 × 5 cm^2^, was mounted on a programmable linear motor system to provide controlled periodic motion. By precisely adjusting the starting position, maximum velocity, and acceleration of the motor, the TENG was subjected to cyclic contact and separation at various frequencies and applied forces. The resulting electrical signals—including open-circuit voltage (V_OC_), short-circuit current (I_SC_), and transferred charge (Q_SC_)—were captured via a high-speed data acquisition system and transmitted to a computer terminal for real-time monitoring. All measurements were conducted using a Keithley 6514 system electrometer (Keithley Instruments, LLC, Beaverton, OR, USA) connected with high-insulation cables under controlled ambient laboratory conditions to ensure data accuracy and reproducibility.

## 3. Results and Discussion

### 3.1. Preparation of Chitosan-Based LIG and LCG Composite Films

**Figure 1:**
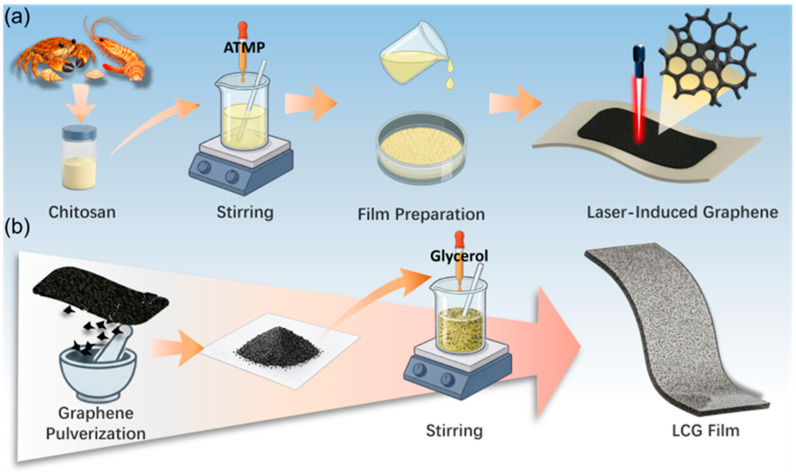
Fabrication process of chitosan-based LIG and LCG composite films. (**a**) One-step synthesis of LIG. (**b**) Preparation of LIG powder and LCG composite films.

As illustrated in Figure 1, the composite films in this study were fabricated using a solution casting method. Initially, chitosan (CS) was dissolved in acetic acid, followed by the addition of a specific amount of ATMP. Specifically, the incorporation of ATMP imparts flame-retardant properties to the composite film. Upon thermal exposure, phosphoric acid species generated from the decomposition of ATMP catalyze the carbonization of the CS matrix, facilitating the formation of a robust intumescent char layer. This char layer acts as a protective barrier that effectively inhibits heat and mass transfer, particularly the diffusion of oxygen and combustible gases, thereby significantly mitigating the structural damage induced by high-intensity laser radiation [26].

The mixture was stirred thoroughly to obtain a homogeneous and stable precursor solution, which was subsequently transferred to a Petri dish and dried in an oven to achieve dehydration and film formation. The as-fabricated films were then subjected to direct laser engraving under ambient conditions using a commercial CO_2_ infrared laser to in situ synthesize LIG (detailed processing parameters are provided in Section 2) (Figure 1a).

To ensure the effective integration of LIG into the polymer matrix, the as-prepared LIG was exfoliated from the substrate and ground into a fine powder (Figure 1b). Subsequently, the LIG powder was dispersed in a 2% *v*/*v* acetic acid solution via ultrasonication for 10 min to prevent aggregation. Chitosan powder and glycerol (acting as a plasticizer) were then sequentially added to the suspension, followed by continuous magnetic stirring in a water bath at 30 °C for 5 h to obtain a homogeneous LCG precursor. Finally, the LCG composite films were obtained by casting the mixture into a mold and drying it at 40 °C.

### 3.2. Characterization of LIG

**Figure 2:**
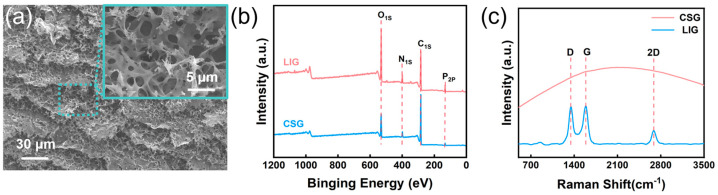
(**a**) SEM image of LIG. (**b**) XPS spectra of CSG and LIG. (**c**) Raman spectra of CSG and LIG.

As illustrated in Figure 2a, a dense 3D graphitic network is observed at a resolution of 30 μm. Consistent with the laser conversion behavior reported for polyimide and other natural substrates, the as-prepared LIG surface manifests characteristic micropores and fissures of varying diameters. It is hypothesized that laser irradiation on the CS matrix induces an instantaneous localized high-temperature and high-pressure environment, which triggers the pyrolytic cleavage of chemical bonds within the CS molecular chains, specifically C-O and C-N [27,28]. This process leads to the rapid evolution of gaseous products, where the internal pressure buildup and the subsequent bursting of these volatile gases through the carbonizing matrix result in the formation of a distinct porous morphology. As illustrated by the XPS characterization in Figure 2b, the elements C, N, O, and P remain present after laser engraving. Further quantitative analysis of the elemental content (Tables S1 and S2) reveals that the carbon atomic percentage increased by 7.99%, while the nitrogen (N) content decreased to 0.21%. These results indicate that during the high-temperature pyrolysis process, nitrogen elements escaped in the form of volatile small molecules due to the cleavage of C-N bonds. As shown in the Raman spectra in Figure 2c, compared with the pure chitosan (CS) spectrum, the laser-induced graphene (LIG) exhibits distinct D, G, and 2D peaks. The I_D_/I_G_ ratio is approximately 0.98, which is consistent with the previously reported range for LIG. This value reflects a disordered graphitic structure with inherent defects produced by the photothermal conversion during the laser induction process [29]. These Raman signatures confirm the successful fabrication of LIG on the ATMP-CS composite film through the one-step CO_2_ laser engraving process [30,31].

### 3.3. Characterization of the Optical and Physical Properties of LCG

**Figure 3:**
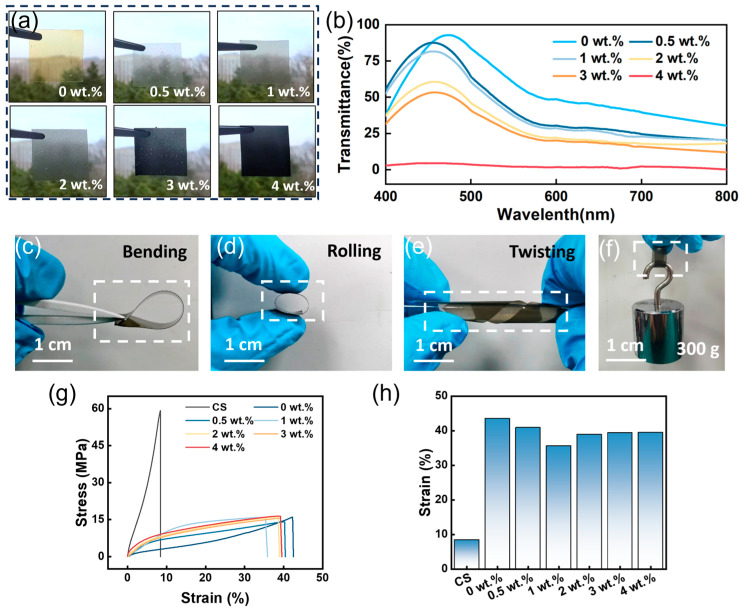
(**a**) Digital photographs of LCG composite films with different LIG loadings. (**b**) Transmittance of LCG composite films with different LIG loadings. (**c**–**f**) Optical photographs of the film under bending, rolling, and twisting. (**g**) Stress–strain curves of pure CS films and LCG composite films; (**h**) Elongation at break of pure CS films and LCG composite films.

As illustrated in Figure 3a, the optical transmittance of the LCG composite films, incorporating various LIG mass fractions (0, 0.5, 1, 2, 3 and 4 wt.%), exhibits a pronounced downward trend as the LIG loading increases. Although the films maintain some degree of clarity across the tested concentration range, a significant reduction in transparency is observed specifically at an LIG concentration of 4 wt.%. Furthermore, as depicted in Figure 3b, the transmittance of the LCG films decreases as the LIG content rises. Notably, a precipitous drop in transmittance occurs at the 4 wt.% threshold. The dispersion quality of LIG fillers was further examined using optical microscopy, as shown in Figure S1. The optical microscopy images reveal that the LIG particles maintain a globally uniform distribution within the CS matrix at concentrations of 1, 2, and 4 wt.%. This high degree of dispersion ensures the spatial uniformity of charge-trapping sites, which is essential for the enhanced electrical performance. While some localized microscopic clusters are observed due to the manual grinding process, these particles are completely encapsulated by the insulating CS matrix. Consequently, they remain isolated without forming continuous conductive pathways, particularly at the optimal 1 wt.% concentration, thereby preserving the overall dielectric strength and charge-storage capacity of the LCG composite films [32].

This experimental finding is highly consistent with the visual evidence provided by the optical photographs in Figure 3d. Figure 3c–f present optical photographs of the as-prepared LCG composite films, with various deformations, including bending, rolling, and twisting, demonstrating its excellent flexibility. As illustrated in Figure 3f, the film is capable of supporting a 300 g weight, thereby verifying the good mechanical robustness of the LCG composite film.

To further characterize the mechanical properties, the stress–strain curves and elongation at break of the pure CS sample (without glycerol) and the LCG composite films with varying LIG contents (containing glycerol) were compared (Figure 3g,h). The pure CS film exhibited an elongation at break of only 8.4%, whereas the samples with glycerol showed a significant increase to approximately 40%. Conversely, while the pure CS film possessed a fracture strength of 59.2 MPa, the addition of glycerol resulted in a reduction in the fracture strength to approximately 15 MPa. This phenomenon can be attributed to the fact that due to its small molecular size and high density of hydroxyl groups, glycerol competes for hydrogen bonding sites, effectively disrupting the inherently strong interchain hydrogen bonds of chitosan. The mitigation of these interchain forces significantly facilitates the segmental mobility of the polymer chains, resulting in a substantial increase in elongation at break and overall flexibility [33]. In addition, the retention of mechanical ductility upon LIG incorporation provides indirect yet compelling evidence for favorable filler–matrix interfacial adhesion. As shown in Figure 3h, the elongation at break of the glycerol-plasticized composites remains at a high level of approximately 40% even after LIG incorporation. In polymer composites, poor interfacial bonding typically induces severe stress concentration at the filler–matrix boundary, leading to premature crack initiation, filler pull-out, and a dramatic reduction in elongation. The fact that the LCG films maintain substantial flexibility with only a marginal decrease in elongation at break therefore indicates that the LIG fillers are well-anchored within the chitosan matrix, enabling homogeneous stress distribution and efficient interfacial load transfer without significant debonding.

### 3.4. Characterization of the Degradation Performance of LCG Composite Films

**Figure 4:**
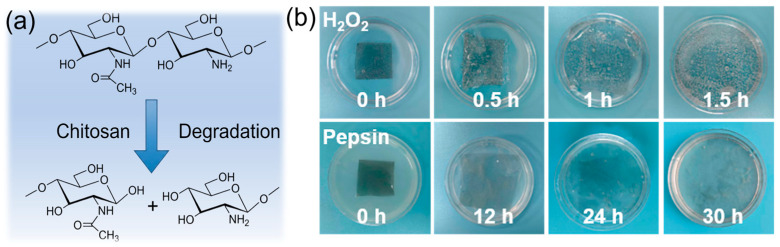
(**a**) Photograph of LCG composite film lifting a weight. (**b**) Optical photographs of the LCG film degrading in hydrogen peroxide solution and pepsin solution.

To validate the superior and comprehensive biodegradability of the material, degradation assessments were extended to various media, including hydrogen peroxide (H_2_O_2_) and enzymatic solutions. The specific molecular architecture of CS, which consists of 2-acetamido-D-glucose and 2-amino-D-glucose units condensed via glycosidic bonds, serves as the structural basis for its environmental responsiveness. As illustrated in Figure 4a, the susceptibility of these glycosidic linkages to pyrolytic or chemical cleavage facilitates the fragmentation of the matrix into oligomers, thereby driving the rapid degradation observed in the composite films. This distinct molecular architecture endows the material with outstanding degradation capabilities across diverse environmental conditions [34]. Degradation experiments in multiple media further confirm the excellent bioenvironmental adaptability of the LCG films. As depicted in Figure 4b, the films underwent significant oxidative degradation when immersed in H_2_O_2_ solution. Attributed to the superior hygroscopicity of CS and the glycerol component, the samples exhibited rapid twisting and conformational distortion upon contact with the solution, displaying noticeable swelling and localized degradation within 0.5 h, and completely disappearing after 1.5 h. Conversely, in a pepsin solution at 37 °C, the films gradually swelled and faded in color over time, achieving complete degradation after 30 h. It is worth emphasizing that although the LIG component remains as a solid residue during the degradation process, this does not contradict the original design intent of green electronic materials. Unlike widely used synthetic substrates such as polyimide (PI) or polyethylene terephthalate (PET), which are environmentally persistent and difficult to recycle, the LCG films in this study reduce the proportion of non-degradable waste to an extremely low level (approximately 1 wt.%). Since the residual LIG originates from the in situ conversion of biomass precursors, it is essentially chemically inert inorganic carbon. Owing to its exceptional stability and inherent environmental benignity, this residue possesses excellent ecological compatibility with both soil and water systems [35,36].

### 3.5. Working Mechanism and Electrical Output Characterization of LCG-TENG

**Figure 5:**
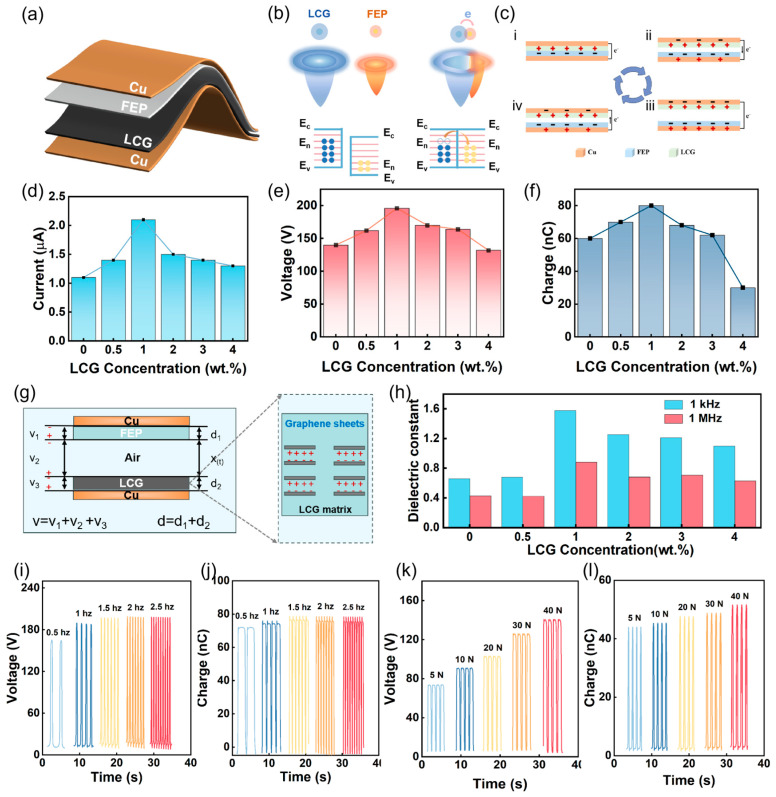
Working mechanism, electrical output performance, and optimization of the LCG-TENG. (**a**) Schematic illustration of the LCG-TENG. (**b**) Electron cloud models and charge transfer processes between LCG and FEP layers. (**c**) Schematic diagram illustrating the working principle of the TENG. (**d**–**f**) Electrical output performance, including (**d**) open-circuit voltage (V_OC_), (**e**) short-circuit current (I_SC_), and (**f**) transferred charge (Q_SC_) of the LCG-TENG with various LIG loadings. (**g**) Schematic of the theoretical dielectric-dielectric capacitance model established between LCG and FEP. (**h**) Relative permittivity of LCG with different LIG loadings at 1 kHz and 1 MHz. (**i**,**j**) Output voltage (**i**) and current (**j**) under various frequencies. (**k**,**l**) Output voltage (**k**) and current (**l**) under various applied forces.

To evaluate the output performance, a contact-separation mode LCG-TENG was developed using the as-prepared composite films. Figure 5a illustrates the structural schematic of the LCG-TENG, where LCG serves as the tribopositive material, fluorinated ethylene propylene (FEP) as the tribonegative material, and copper (Cu) as the electrode. Figure 5b provides a detailed elucidation of the electron cloud distribution and charge transfer behavior between LCG and FEP at the atomic level. Prior to physical contact, the electron clouds of LCG and FEP remain in independent states, with electrons confined within their respective potential wells in specific surface states. According to the electron-cloud-potential-well model, when LCG and FEP come into physical contact, the electron clouds of the two materials significantly overlap, leading to a reduction in the potential barrier for charge transition at the interface. Since the biomass-derived LCG possesses a lower work function, its surface energy level is higher than that of the more electronegative FEP. Upon separation, as the interatomic distance increases, the potential barrier rapidly restores and prevents charge back-flow, allowing most of the transferred electrons to be retained on the FEP surface. Consequently, this results in a net positive electrostatic charge on the LCG surface and a net negative charge on the FEP surface [37,38].

Figure 5c illustrates the operational mechanism of the developed TENG under the synergistic coupling of triboelectrification and electrostatic induction. In the initial separated state, no potential difference exists between the FEP film and the LCG film. When an external force drives the positive and negative triboelectric layers into direct contact, positive charges accumulate on the LCG surface, while an equivalent amount of negative charges builds up on the FEP film. Subsequently, as the two layers separate, electrons migrate through the external circuit from the top Cu electrode to the electrode fixed at the bottom of the FEP film to effectively screen the electrostatic field generated by the surface-bound charges, thereby producing a forward current. The current drops to zero once the separation distance reaches its maximum. Conversely, when the triboelectric layers approach each other again, electrons flow in the reverse direction to maintain the electric field equilibrium between the positive and negative charges, resulting in a reverse current [39,40,41].

As depicted in Figure 5d, the open-circuit voltage (V_OC_) progressively increases with LIG content, rising from 140 V to a peak value of 196 V. Similarly, the short-circuit current (I_SC_) shown in Figure 5e increases from 1.1 μA to a maximum of 2.1 μA. The transferred charge (illustrated in Figure 5f) also increases from 60 nC to a peak of 82 nC. These results indicate that the output performance of the TENG exhibits an initial enhancement followed by a subsequent decline, reaching its optimal output at an LIG concentration of 1 wt.%. The underlying mechanism for this enhancement is illustrated in Figure 5g. During continuous contact-separation cycles, triboelectric charges are initially generated and accumulated on the surface of the LCG composite film. Subsequently, these surface charges penetrate the triboelectric layer and are captured by charge-trapping sites formed by the dispersed LIG particles. These incorporated LIG particles function as a distributed micro-capacitor network within the CS matrix, which significantly facilitates efficient charge transport and storage. This unique structural configuration enables the seamless transfer of triboelectric charges from the material surface to internal storage sites, effectively ensuring the device’s exceptional long-term charge retention and output stability. The introduction of LIG optimizes the dielectric characteristics of the triboelectric layer, minimizing internal resistance losses while concurrently reinforcing the capacitive effect of the device. To quantitatively evaluate its contribution to the output gain, a theoretical capacitance model was constructed by referencing prior research, systematically deconstructing the energy conversion process of the LCG-TENG [42,43]. While the actual operation of TENGs involves complex non-ideal factors, the device is modeled as an ideal capacitor under simplified conditions to focus on the primary electrostatic interactions.

The LCG-TENG is equivalent to an ideal capacitor, where FEP and LCG (with relative dielectric constants ε1 and ε2, respectively) serve as the negative and positive friction layers. The separation distance X_(t)_ between the two layers varies periodically with the operation of the LCG-TENG. It should be acknowledged that this model represents an idealized theoretical frame-work; however, it remains the most foundational and widely adopted approach in the field, pioneered by Prof. Z.L. Wang and colleagues, providing an essential tool for evaluating the relationship between surface charge density (σ), displacement, and electrical output [44]. Upon contact, the two triboelectric layers acquire equal and opposite charge densities (σ). During the subsequent separation, an increasing X_(t)_ generates a potential difference (V) between the two electrodes. Based on electrodynamic derivations, the V-X_(t)_ relationship is expressed as follows:

(1) $$V\;=\;E_{1}d_{1\;}+\;E_{2}d_{2\;}+\;E_{\mathrm{air}}X_{\left(t\right)}\;=\;-\frac{Q}{S\varepsilon_{0}}\left(d_{0}\;+\;X_{\left(t\right)}\right)\;+\;\frac{\sigma X_{\left(t\right)}}{\varepsilon_{0}}$$

where S, ε_0_, σ, and d_0_ denote the contact area, vacuum permittivity, surface charge density, and effective dielectric thickness, respectively. Based on Equation (1), the expressions for the open-circuit voltage (V_OC_), short-circuit current (I_SC_), and transferred surface charge (Q_SC_) can be derived as follows:

(2) $$V_{\mathrm{OC}}=\frac{\sigma X_{\left(t\right)}}{\varepsilon_{0}}$$

(3) $$Q_{\mathrm{SC}}=\frac{S\sigma X_{\left(t\right)}}{X_{\left(t\right)}+d_{0}}$$

(4) $$I_{\mathrm{SC}}=\frac{dQ_{\left(\mathrm{SC}\right)}}{\mathrm{dt}}=\frac{S\sigma d_{0}}{{\left(d_{0}+X_{\left(t\right)}\right)}^{2}}\frac{d_{x}}{d_{t}}$$

As indicated by the aforementioned equations, the outputs V_OC_, I_SC_, and Q_SC_ are all fundamentally governed by the surface charge density (σ) of the dielectric layer and the separation distance X_(t)_. As established earlier, the TENG is analogous to an ideal capacitor, and its capacitance can be described by the following expression:

(5) $$C=\frac{\varepsilon_{r}S}{4\Pi \mathrm{Kd}}=\frac{\sigma S}{\mathrm{Ed}}$$

This implies that at a constant thickness (d) and contact area (S), the surface charge density (σ) is directly proportional to the relative dielectric constant (ε) of the dielectric layer. Consequently, augmenting the dielectric properties of the composite material will significantly enhance the output performance of the TENG. As illustrated in Figure 5h, the frequency-dependent dielectric properties of both pure CS and LCG films were investigated at room temperature within a frequency range of 10^3^ to 10^6^ Hz. Throughout the entire measurement spectrum, the dielectric constants of the LCG films remained consistently higher than those of the pure CS films. Furthermore, with the increase in LIG loading, the dielectric constant of the LCG films exhibited a trend of initially increasing and subsequently decreasing. To further investigate the enhancement mechanism of LIG fillers on the performance of the triboelectric nanogenerator, the dielectric constant was first characterized, as shown in Figure 5h. The results demonstrate that the incorporation of LIG significantly elevates the dielectric permittivity of the composite film. This dielectric enhancement facilitates a higher surface charge density, thereby promoting efficient charge transfer during contact-separation cycles. Furthermore, owing to its unique inherent porous architecture, the LIG filler enables the construction of a micro-capacitor network within the polymer matrix. This network functions as effective charge traps to capture and store induced triboelectric charges, significantly inhibiting charge dissipation and ensuring long-term charge retention. However, a decline in output performance is observed when the LIG concentration exceeds 1 wt.%. This is primarily attributed to the formation of continuous conductive pathways between LIG particles, which accelerates charge dissipation and reduces effective charge storage, ultimately degrading the device performance [45].

The power generation performance of the LCG film was evaluated under varying frequencies and applied pressures. The results demonstrate that the V_OC_ (Figure 5i) and Q_SC_ (Figure 5j) do not exhibit significant variations as the frequency increases. In contrast, the short-circuit current (I_SC_) shows a positive correlation with frequency (Figure S2). At a frequency of 2.5 Hz, the film attained its maximum V_OC_ of 196 V. Furthermore, the V_OC_ increases with the rising external pressure, reaching 139 V under an applied pressure of 40 N (Figure 5k). Both Q_SC_ and I_SC_ followed the same trend as V_OC_ (Figure 5l and Figure S3). These observed trends are attributed to the accelerated induction and transfer of charges under high-frequency contact conditions.

### 3.6. Energy Harvesting Applications of the LCG-TENG

**Figure 6:**
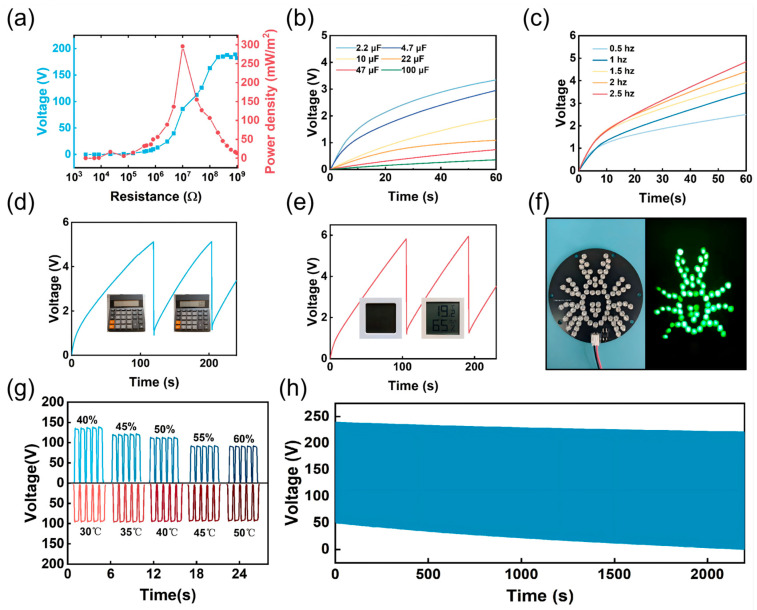
Practical applications, environmental stability, and long-term reliability of the LCG-TENG. (**a**) Output voltage and power density of the TENG as a function of external load resistance. (**b**) Charging curves of capacitors with different capacitances. (**c**) Charging curves of a specific capacitor under various frequencies. (**d**,**e**) Digital calculator (**d**) and hygrothermograph (**e**) powered by a 4.7 μF capacitor charged by the TENG. (**f**) Photograph of spider-shaped LEDs illuminated by the LCG-TENG. (**g**) Comparison of output stability of the LCG-TENG under various humidity and temperature conditions. (**h**) Cyclic stability test of the LCG-TENG over 2300 cycles.

As illustrated in Figure 6a, the output characteristics of the TENG relative to the load resistance were investigated under peak output conditions. By employing a parallel configuration with various external resistors, the dependence of the output voltage on the load resistance was systematically evaluated. The results demonstrate that the output voltage increases monotonically as the external load resistance rises. Furthermore, the power density (P) of the TENG was determined according to Equation:

(6) $$P=\frac{U^{2}}{\mathrm{RA}}$$

where U denotes the output voltage across the load resistance R, and A represents the effective contact area. The output power of the LCG-TENG initially rises and subsequently declines, reaching a peak power density of 295 mW/m^2^. To evaluate the charging capacity of the LCG-TENG, it was connected to a series of commercial capacitors with distinct capacities (2.2, 4.7, 10, 22, 47, and 100 μF) through a full-wave rectifier bridge. The real-time output voltage profiles across these capacitors were recorded to systematically investigate the energy storage efficiency. As shown in Figure 6b, capacitors with lower capacitance exhibited higher terminal voltages within the same charging interval, exhibiting the robust energy harvesting capability of the TENG. Figure 6c illustrates the charging performance for a 2.2 μF capacitor at different contact frequencies. The charging duration significantly decreases as the frequency increases, with the voltage approaching 5V within 60 s at a frequency of 2.5 Hz.

With excellent charging performance, the LCG-TENG can harvest mechanical energy to power small electronic devices. As shown in Figure 6d,e, the LCG-TENG was applied to charge a 4.7 μF capacitor to drive a calculator and a digital thermo-hygrometer. Furthermore, the LCG-TENG successfully illuminated a spider-shaped LED array (Figure 6f). The environmental stability of the LCG-TENG was also investigated, as depicted in Figure 6g. The output voltage remained consistent across a temperature range of 30 to 50 °C, while a marginal decline was observed as the relative humidity increased from 40% to 70%. This phenomenon is primarily attributed to the fact that chitosan is a natural polysaccharide containing abundant hydroxyl (-OH) and amino (-NH_2_) groups, which render chitosan highly hygroscopic. When environmental humidity increases, the adsorbed water molecular layer leads to surface charge dissipation. Additionally, Figure 6h presents the cyclic stability of the LCG-TENG over a 2300 s duration at a contact frequency of 1 Hz. The LCG-TENG maintained remarkably stable triboelectric output throughout the contact-separation cycles, consistently delivering a high output of approximately 200 V even after prolonged operation.

### 3.7. Real-Time Sensing of Human Motion via LCG-TENG

By adhering the LCG-TENG to different anatomical sites, it is possible to monitor a wide range of physiological signals and physical movement states.

**Figure 7:**
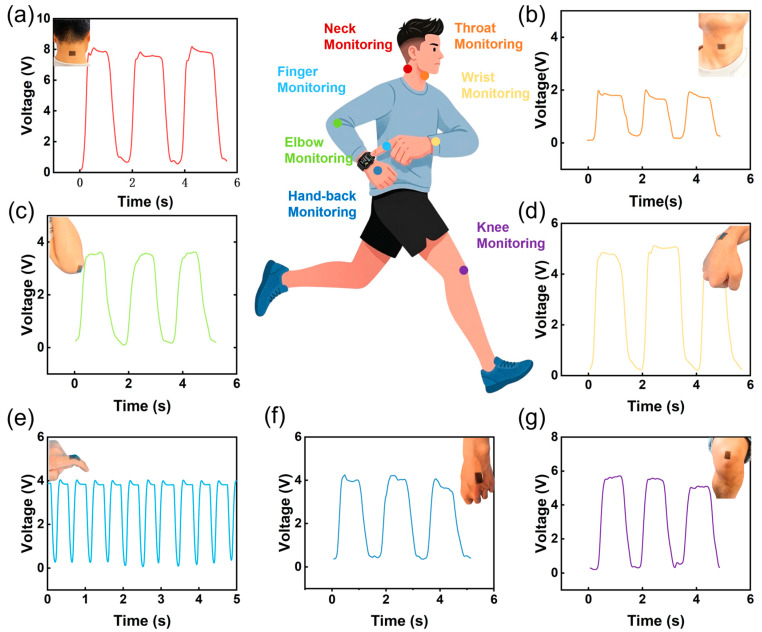
Practical applications of the LCG-TENG as a self-powered wearable sensor for real-time human motion monitoring. (**a**) Neck bending, (**b**) Throat movement (swallowing/vocalization), (**c**) Elbow bending, (**d**) Wrist bending, (**e**) Finger bending, (**f**) Hand back movement (tapping/clenching), and (**g**) Knee bending.

Figure 7a–g display the electrical signals generated by the LCG-TENG sensor during movements across various body parts, including the neck, throat, elbow, wrist, finger, and knee. As illustrated in Figure 7a, the LCG-TENG sensor directly produces electrical signals during neck bending, exhibiting excellent repeatability and stability. Furthermore, when the finger (Figure 7e) or knee (Figure 7g) undergoes bending, the resulting contact and separation between the skin and the sensor generate a distinct and measurable electrical output.

## 4. Conclusions

In summary, this study proposes an innovative strategy of modifying chitosan (CS) with ATMP for the successful fabrication of 3D porous LIG via a one-step CO_2_ laser direct-writing process. By incorporating the synthesized LIG powder as a functional filler, high-performance and biodegradable TENGs were further developed. Experimental results demonstrate that the device achieves a V_OC_ of 196 V and a power density of 295 mW/m^2^, which is attributed to the distributed micro-capacitor network formed by LIG within the matrix that significantly enhances the charge storage capacity and dielectric characteristics. Furthermore, the composite film exhibits exceptional environmental sustainability, undergoing complete biodegradation within 30 h. More importantly, the self-powered sensors based on LCG-TENG enable real-time, precise, and stable monitoring of human motions, including neck, finger, and knee movements. This research provides a new perspective for the green production of biomass-derived graphene and holds broad application prospects in the fields of sustainable flexible electronic skins and green wearable sensing.

## Figures and Tables

**Table 1:** Composition and Fabrication Parameters of CSG and LCG Composite Films.

Sample Label	Chitosan (CS) (g)	Glycerol (g)	LIG (wt.%)
CSG	1.0	0.3	0
LCG-0.5	1.0	0.3	0.5
LCG-1	1.0	0.3	1
LCG-2	1.0	0.3	2
LCG-3	1.0	0.3	3
LCG-4	1.0	0.3	4

## Data Availability

The original contributions presented in this study are included in the article/Supplementary Materials. Further inquiries can be directed to the corresponding author.

## Supplementary Materials

The following supporting information can be downloaded at: https://www.mdpi.com/article/10.3390/nano16090550/s1, Figure S1: Optical microscopy images of LCG composite films with LIG concentrations of (a) 0 wt.%, (b) 1 wt.%, (c) 2 wt.%, and (d) 4 wt.%. Figure S2: Output current at different frequencies. Figure S3: Output current under various applied forces. Table S1: Elemental analysis of the laser-induced graphene (LIG) surface derived from ATMP-modified chitosan. Table S2: Elemental analysis of the ATMP-modified chitosan (CSG) composite film (unengraved substrate).
