# Supplementary material for: Biomass-Derived Laser-Induced Graphene/Chitosan Composite Films for Sustainable Triboelectric Nanogenerators
Source: Nanomaterials (Basel). 2026 Apr 30;16(9):550. doi: 10.3390/nano16090550 (PMC13165254; doi:10.3390/nano16090550)
Supplement: Supplementary file 1 [file nanomaterials-16-00550-s001.zip › nanomaterials-4259142-supplementary.pdf]

Table S1. Elemental analysis of the laser-induced graphene (LIG) surface derived from ATMP-modified chitosan.

Element	Atomic %
C	77.57
N	0.21
O	21.21
P	1.01

Table S2 Elemental analysis of the ATMP-modified chitosan (CSG) composite film (un-engraved substrate)

Element	Atomic %
C	69.58
N	4.39
O	21.41
P	4.62

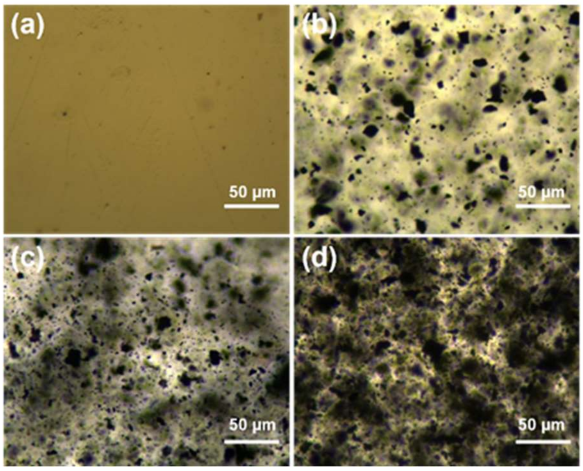

Figure S1. Optical microscopy images of LCG composite films with LIG concentrations of (a) 0 wt.%, (b) 1 wt.%, (c) 2 wt.%, and (d) 4 wt.%.

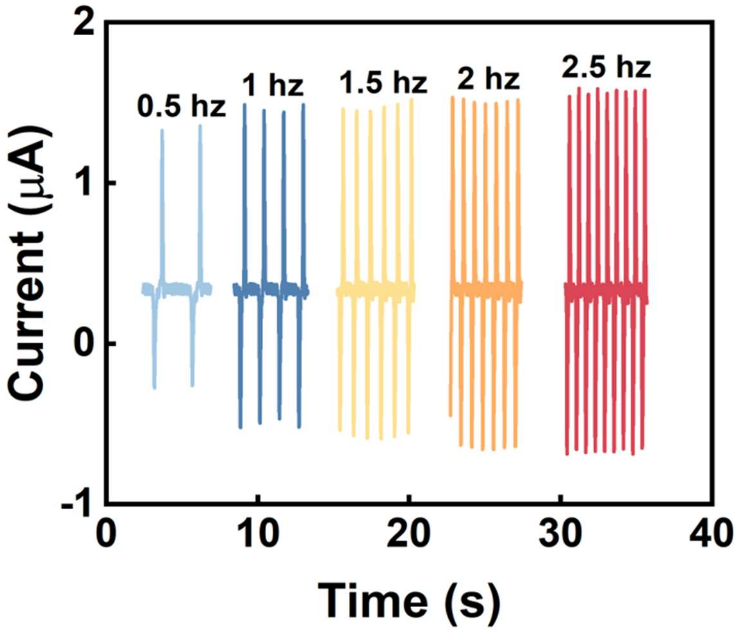

Figure S2. Output current at different frequencies

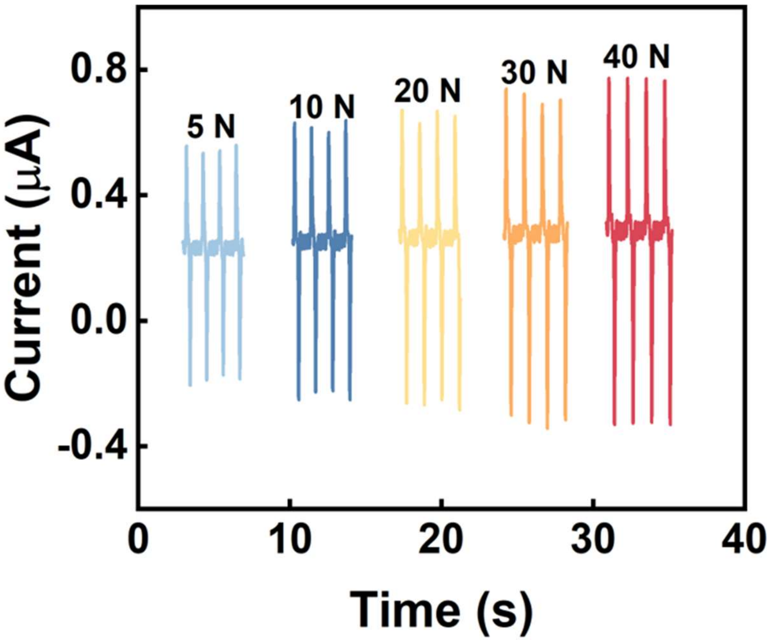

Figure S3. Output current under various applied forces
